# You can have your breastmilk and safe sleep too: a preliminary analysis of infant safe sleep data in a Midwestern home visiting program

**DOI:** 10.1186/s40621-018-0138-y

**Published:** 2018-04-10

**Authors:** Sheena Hussain, Gina S. Lowell, Douglas R. Roehler, Kyran P. Quinlan, S. Darius Tandon, Lesley Schwartz

**Affiliations:** 1 Chicago Medical School, Rosalind Franklin University of Medicine and Science, North Chicago, IL USA; 2 Department of Pediatrics, Rush University Children’s Hospital, Chicago, IL USA; 3 Center for Community Health, Northwestern University Feinberg School of Medicine, Chicago, IL USA; 4 The Maternal, Infant, and Early Childhood Home Visiting (MIECHV) Program, Illinois’ Governor’s Office of Early Childhood Development (OECD), Chicago, IL USA

**Keywords:** Infant safe sleep, Breastfeeding, Household tobacco use, Home visiting

## Abstract

**Background:**

Sudden unexpected infant death (SUID) accounted for approximately 3700 infant deaths in the US in 2015. SUID risk factors include prone sleeping, bed-sharing, soft bedding use, and maternal smoking. Infant safe sleep data in at-risk communities are difficult to obtain and home visiting programs can add to what we know. This study’s purpose is to determine how often caregivers enrolled in home visiting programs provide safe sleep environments for their infants in relation to breastfeeding status and tobacco use.

**Methods:**

Female caregivers in at-risk communities were prospectively enrolled in Midwestern home visiting programs. Those that had infants < 365 days old and completed a safe sleep survey between October 1, 2016 and May 18, 2017 were included. Caregivers’ responses (always, sometimes, or never) to three safe sleep questions were compared by breastfeeding status, caregiver tobacco use, and household tobacco use using Pearson’s chi-squared or Fisher’s exact test.

**Results:**

The characteristics of the 289 eligible female caregivers included 120 (42%) ≤ 21 years old, 137 (47%) black, 77 (27%) breastfeeding, and 60 (22%) with household tobacco use. Two hundred forty-six (85%) caregivers always placed infants in the supine position, 148 (51%) never bed-shared, and 186 (64%) never used soft bedding. Ongoing breastfeeding caregivers never bed-shared more often than those who never breastfed or weaned (66% vs. 53% vs. 39%, *p* = 0.003). Households with tobacco use placed infants in the supine position less (75% vs. 88%, *p* = 0.03), bed-shared more (62% vs. 44%, *p* = 0.04), and used soft bedding more (50% vs. 32%, *p* = 0.004) relative to those without tobacco use.

**Conclusions:**

In this group of at-risk young mothers, those who breastfed bed-shared less than mothers who were not breastfeeding; this finding has implications toward reducing the SUID risk in similar populations. This study also demonstrated that infants living with a tobacco user are less likely to be sleeping safely. This suggests that a multifaceted approach to safe sleep counseling may be needed.

## Background

Despite a 40% reduction in sudden unexpected infant deaths (SUID) since the 1990s, there were still over 3700 SUIDs in the United States during 2015, and this rate has remained unchanged for several years (Sudden Unexpected Infant Death and Sudden Infant Death Syndrome Data and Statistics 2016). SUID, which is defined as any sudden or unexpected death of an infant 12 months or younger, can be further divided into three categories: sudden infant death syndrome (SIDS) comprising 43%, unknown causes comprising 32%, and accidental suffocation and strangulation in bed comprising 25% (Sudden Unexpected Infant Death and Sudden Infant Death Syndrome Data and Statistics 2016).

Providing a safe sleep environment for infants can help reduce the stagnant SUID rate. The American Academy of Pediatrics (AAP) has been releasing safe sleep guidelines for over two decades, and it currently provides 19 recommendations that identify SUID risk and protective factors. Risk factors include prone sleeping, bed-sharing, soft bedding use (blankets, pillows, soft toys, loose sheets), and maternal smoking. An identified protective factor is breastfeeding (Moon and Task Force On Sudden Infant Death 2016). The AAP’s safe sleep recommendations, however, are not without controversy; a well-known tension exists between promoting breastfeeding and promoting safe sleep. Although breastfeeding is protective against SUIDs, many breastfeeding mothers also bed-share, a common risk factor for SUIDs. Research studies have found a positive association between bed-sharing and breastfeeding worldwide, and many groups believe that bed-sharing is a necessary tool for facilitating successful breastfeeding (Thoman 2006; Horsley et al. 2007; Mileva-Seitz et al. 2016; McKenna and Gettler 2016; Ball 2003; Ward 2015). While the AAP promotes breastfeeding, they emphatically warn that bed-sharing can increase the risk of infant strangulation, suffocation, entrapment, overlay, and other unintentional injuries (Moon and Task Force On Sudden Infant Death 2016; Mileva-Seitz et al. 2016). The AAP does recommend room-sharing without bed-sharing for the first year of life.

Many groups believe that safe sleep messages and interventions should be targeted to those most at-risk for SUIDs, given how little progress has been made over the past several years. Caregivers who are single, have a low economic status, young maternal age, and low education level are at a higher risk for SUID occurrence along with infants less than 4 months old, premature infants, and infants with a low birth weight (Moon and Task Force On Sudden Infant Death 2016; Athanasakis et al. 2011). Additionally, SUID disproportionately affects non-Hispanic black and Native American infants who have twice the risk of SUID than non-Hispanic white infants (Moon and Task Force On Sudden Infant Death 2016). However, data assessing safe sleep behaviors in these high-SUID risk groups are difficult to obtain. Home visiting programs can add to what we know about safe sleep in these at-risk populations. Typically at-risk communities are defined as areas with “high concentration of specific social dilemmas and negative outcomes for children including premature birth, low-birth weight infants, infant mortality, particularly early death due to child maltreatment, poverty, crime, domestic violence, high rates of school drop-outs, substance abuse, unemployment, and child maltreatment” (Deborah Daro et al. 2010). This definition of at-risk overlaps with many of the SUID risk factors. The Maternal, Infant, and Early Childhood Home Visiting (MIECHV) program provides grants to support evidence-based, voluntary home visiting programs for low-income pregnant women in at-risk communities nationwide (Home Visiting 2017). As of October 2016, Illinois MIECHV began assessing infant safe sleep behaviors, allowing a look into safe sleep practices of high-risk groups.

The purpose of this study is to describe and analyze female caregivers served by Illinois MIECHV-supported home visiting programs to determine and compare their safe sleep behaviors by breastfeeding status and tobacco use. Given the amalgam of risk factors that define this population, we hypothesize that in line with other studies, mothers who breastfeed will have higher rates of bed-sharing compared to those who are not breastfeeding. Additionally, we hypothesize that tobacco users will practice safe sleep less often compared to those who do not use tobacco. This analysis will serve to inform the development of comprehensive safe sleep trainings and policies for home visitors and caregivers, which can help reduce the SUID risk of infants in these populations.

## Methods

### Sample

This was a prospective study of at-risk female caregivers participating in an Illinois MIECHV-supported home visiting program who completed a safe sleep survey from October 1, 2016 to May 18, 2017. Data were from 19 home visiting programs and 6 doula programs within 13 at-risk counties and community areas (i.e., Cicero, Englewood, Rockford, Elgin, Vermillion, Macon, Mid-Central, DeKalb, Stephenson, East St. Louis, Kankakee, Peoria and Austin/North Lawndale).

To be eligible for inclusion in the analyses, female caregivers had to complete at least one safe sleep survey for an infant who was younger than 365 days at the time of the survey. If multiple safe sleep surveys existed for the same infant, only the most recent entry was included. Exclusion criteria included caregivers who completed safe sleep surveys for children older than 12 months and male caregivers. Specifically, male caregivers were excluded due to them making up only 1% of the sample; this made the sample more homogenous.

### Measuring safe sleep responses, breastfeeding status, and tobacco use

Safe sleep questions were added to MIECHV collecting forms on October 1, 2016. The three safe sleep questions were: (SS1) how often is your baby placed to sleep on his/her back, (SS2) how often does your baby bed-share with you or anyone else, (SS3) and how often does your baby sleep with soft bedding (Illinois Maternal 2016). Of the three response choices (always, sometimes, and never), a parent was considered to be practicing safe sleep if they self-reported (SS1) as “always,” and (SS2) and (SS3) as “never.” Furthermore, these questions did not differentiate between napping and nighttime sleep, and was assumed to include all types of sleep. Home visitors were also not required to observationally confirm caregivers’ responses. Home visitors entered the caregiver’s responses for the three safe sleep questions into the MIECHV database, VisitTracker (https://www.visittrackerweb.com/). All relevant data from these surveys were extracted in a de-identified, aggregate manner from VisitTracker. The University of Illinois, Urbana-Champaign Institutional Review Board approved this study under the Maternal, Infant, and Early Childhood Home Visiting Program Evaluation.

The safe sleep survey questions were compared by breastfeeding status, caregiver tobacco use, and household tobacco use. Caregiver tobacco use was extracted from the MIECHV data collection form for primary caregivers (MIECHV data collection form for primary caregiver 2016) while household tobacco use and breastfeeding status were extracted from forms measuring the six MIECHV benchmarks (Illinois Maternal 2016).

### Statistical analyses

The associations between safe sleep survey responses and breastfeeding status, caregiver tobacco use, and household tobacco use were analyzed using Fisher’s exact test or Pearson’s chi-squared test. Fisher’s exact test was used if the sample size in any cell was less than five; otherwise, Pearson’s chi-squared test was used. Associations were considered significant if they had a *p* ≤ 0.05. All data analyses were performed using STATA, version 14.2. (StataCorp, College Station, TX).

## Results

Between October 1, 2016 and May 18, 2017, 567 safe sleep surveys were completed by caregivers. Of the 567 responses, 207 were excluded from this study because they were completed for infants prenatally or infants older than 364 days. Of the remaining 360 survey responses, 68 were excluded because caregivers had completed the sleep survey more than once for their infant. An additional three responses from male caregivers were excluded. Therefore, 289 unique survey responses from female MIECHV caregivers who had infants less than 365 days old at the time of the survey were included in this study.

A description of the caregiver population can be found in Table 1. Of the 289 caregiver respondents, 120 (42%) were 21 years old or younger, 137 (47%) were African American, and 79 (27%) were Hispanic or Latino. Additionally, 190 (73%) caregivers had a high school education or less and 209 (72%) were never married. In regard to infants, 163 (56%) surveys were completed for infants between 0 and 120 days. Of the variables of interest, 35 (13%) caregivers used tobacco, 60 (22%) reported household tobacco use, and 77 (27%) were currently breastfeeding.

**Table 1:** Characteristics of Study Population

Population Characteristics	*n* (%)
Caregiver Age (289 responses)	
≤ 21 years old	120 (42)
22–25 years old	82 (28)
≥ 26 years old	87 (30)
Caregiver Race (289 responses)	
Black/African American	137 (47)
More Than One Race	43 (15)
White	100 (35)
Other	9 (3)
Caregiver Ethnicity (289 responses)	
Non-Hispanic/Latino	210 (73)
Hispanic/Latino	79 (27)
Caregiver Marital Status (289 responses)	
Never Married	209 (72)
Married	52 (18)
Other	28 (10)
Caregiver Education Level (260 responses)	
Less than High School	78 (30)
High School/GED	112 (43)
Some College +	70 (27)
Caregiver Tobacco Use (274 responses)	
Caregiver Tobacco Use	35 (13)
Household Tobacco Use (270 responses)	
Household Tobacco Use	60 (22)
Maternal Depression (237 responses)	
Depression	31 (13)
Breastfeeding Status (287 responses)	
Never	90 (31)
Weaned	120 (42)
Ongoing	77 (27)
Infant Age (289 responses)	
0–120 days	163 (56)
121–240 days	55 (19)
241–364 days	71 (25)
Practicing Back to Sleep (289 responses)	
Never	5 (2)
Sometimes	38 (13)
Always	246 (85)
Bed-Sharing (289 responses)	
Never	148 (51)
Sometimes	99 (34)
Always	42 (15)
Soft Bedding Use (289 responses)	
Never	186 (64)
Sometimes	58 (20)
Always	45 (16)

### Safe sleep benchmark 1 – Practiced back to sleep

Eighty-five percent (*n* = 246) of respondents stated that they always placed infants to sleep on their back. Table 2 shows safe sleep survey responses by breastfeeding status, caregiver tobacco use, and household tobacco use. Although practicing back to sleep was not significantly associated with breastfeeding status (*p* = 0.35), it was associated with caregiver and household tobacco use (*p* = 0.009 and *p* = 0.03, respectively). Caregivers that did not use tobacco were more likely to practice back to sleep, with 210 (88%) practicing back to sleep compared to 24 (69%) that used tobacco (*p* = 0.009). For households without tobacco present, 185 (88%) caregivers always placed infants on their backs to sleep compared to 45 (75%) with household tobacco use present (*p* = 0.03).

**Table 2:** Safe Sleep Responses by Breastfeeding Status, Caregiver Tobacco Use, and Household Tobacco Use

	Placed Infant on Back to Sleep^b^				Bed-Shared^a^				Used Soft Bedding^a^				
	Never	Sometimes	Always	*p*-value†	Never	Sometimes	Always	*p*-value†	Never	Sometimes	Always	*p*-value†	Total
	*n* (%)	*n* (%)	*n* (%)		*n* (%)	*n* (%)	*n* (%)		*n* (%)	*n* (%)	*n* (%)		*n*
Breastfeeding Status													
Never	1 (1)	16 (18)	73 (81)	0.35	48 (53)	27 (30)	15 (17)	0.003*	51 (57)	22 (24)	17 (19)	0.06	90
Weaned	3 (3)	16 (13)	101 (84)		47 (39)	55 (46)	18 (15)		73 (61)	27 (23)	20 (17)		120
Ongoing	1 (1)	6 (8)	70 (91)		51 (66)	17 (22)	9 (12)		60 (78)	9 (12)	8 (10)		77
Caregiver Tobacco Use													
No	4 (2)	25 (10)	210 (88)	0.009*	127 (53)	79 (33)	33 (14)	0.52	159 (67)	44 (18)	36 (15)	0.15	239
Yes	1 (3)	10 (29)	24 (69)		15 (43)	14 (40)	6 (17)		18 (51)	11 (31)	6 (17)		35
Household Tobacco Use													
No	3 (1)	22 (10)	185 (88)	0.03*	118 (56)	65 (31)	27 (13)	0.04*	143 (68)	33 (16)	34 (16)	0.004*	210
Yes	2 (3)	13 (22)	45 (75)		23 (38)	24 (40)	13 (22)		30 (50)	21 (35)	9 (15)		60

^*^p ≤ .05

^†^Pearson’s chi-squared or Fisher’s exact test were used to determine association

^a^Comparison performed using Pearson’s chi-squared test

^b^Comparison performed using Fisher’s exact test

### Safe sleep benchmark 2 – Bed-shared

Forty-nine percent (*n* = 141) of caregivers reported sometimes or always bed-sharing. Breastfeeding and household tobacco use were significantly associated with bed-sharing (*p* = 0.003 and *p* = 0.04, respectively). Ongoing breastfeeding caregivers were more likely to never bed-share, with 51 (66%) stating they never bed-shared compared to 48 (53%) who never breastfed and 47 (39%) who weaned (*p* = 0.003). Of the 60 caregivers who reported household tobacco use, 37 (62%) reported sometimes or always bed-sharing, compared to 92 (44%) of 210 caregivers who had no household tobacco use (*p* = 0.04).

### Safe sleep benchmark 3 – Used soft bedding

Thirty-six percent (*n* = 103) of caregivers reported that they sometimes or always used soft bedding. Household tobacco use was significantly associated with soft bedding use, with 30 (50%) households with tobacco use sometimes or always using soft bedding compared to 67 (32%) households without tobacco use (*p* = 0.004).

## Discussion

In our study population, we demonstrate the following: breastfeeding caregivers were less likely to bed-share, and households with tobacco use were more likely to place infants in the prone position, bed-share, and use soft bedding. This study demonstrates the capacity of successfully breastfeeding mothers to practice safe sleep, with two-thirds of breastfeeding women never bed-sharing. It also identifies that households with tobacco use have a confluence of risk factors present.

This strong association of breastfeeding without bed-sharing is counter to the evidence-supported view that bed-sharing promotes successful breastfeeding; these results have implications toward providing a more consistent safe sleep message in the future. Generally, there seems to be two popular, yet conflicting safe sleep messages: one that recommends breastfeeding while bed-sharing (McKenna and Gettler 2016) and the other that recommends breastfeeding while room-sharing (Moon and Task Force On Sudden Infant Death 2016). In regards to bed-sharing and breastfeeding, most studies have found a positive association between bed-sharing and breastfeeding worldwide (Thoman 2006; Horsley et al. 2007; Mileva-Seitz et al. 2016; McKenna and Gettler 2016; Ball 2003; Ward 2015), although there is little evidence that shows the temporality between these two behaviors. Some of the benefits of breastfeeding and bed-sharing include physical and emotional mother-infant bonding, better sleep for infants and mothers, and double or triple the number of breastfeeds per night (Mileva-Seitz et al. 2016; McKenna and Gettler 2016; Ward 2015). Some groups therefore believe that bed-sharing is necessary for facilitating successful breastfeeding. Since many mothers tend to breastfeed and bed-share, some groups suggest that safe-sleep messaging should focus on harm reduction, where breastfeeding and bed-sharing occur in safer sleep environments (Thoman 2006; McKenna and Gettler 2016; Ball 2003; Ward 2015).

On the other hand, most pediatricians and researchers, including the AAP, believe that promoting bed-sharing is not necessary for facilitating successful breastfeeding (Moon and Task Force On Sudden Infant Death 2016). In our high SUID risk population, we found that breastfeeding and practicing safe sleep is feasible. This can have implications towards reducing the SUID risk in similar populations. In addition to the results of our study, some groups have also found that many breastfeeding mothers are not bed-sharing. One study of 1194 female caregivers with similar demographics to our population (single, African American caregivers with lower education levels) found that many breastfeeding mothers room-shared, but not bed-shared with their infants (Moon et al. 2017). We hypothesize that women who breastfeed, but not bed-share do so because those who practice positive health behaviors are more likely to practice other positive health behaviors. However, future qualitative studies can be done to understand why some caregivers decide to breastfeed and not bed-share.

Studies like these are first steps toward dismantling the impression that bed-sharing is necessary for facilitating successful breastfeeding. Preliminary safe sleep messages can encourage parents to breastfeed and room-share, without worrying about decreasing breastfeeding rates. Studies that have advised parents to avoid bed-sharing did not see changes in feeding practices (Moon et al. 2017; Smith et al. 2016). However, if parents end up choosing to breastfeed and bed-share, harm reduction is necessary.

Our second finding that household tobacco use was significantly associated with lower safe sleep adherence highlights a new risk factor for unsafe sleep. Most research studies have shown how caregiver tobacco use, both prenatally and postnatally, significantly increases the risk for SUIDs (Moon and Task Force On Sudden Infant Death 2016; Anderson and Cook 1997; Mitchell et al. 1993; Haglund and Cnattingius 1990; Schoendorf and Kiely 1992; Blair et al. 1996; Klonoff-Cohen et al. 1995; Fleming and Blair 2007). Although fewer studies discuss household tobacco use, they have found a dose-response relationship between SUID risk and number of household smokers, number of cigarettes smoked per day, and number of hours of infant exposure to smoke (Haglund and Cnattingius 1990; Klonoff-Cohen et al. 1995; Fleming and Blair 2007). Addressing household tobacco use may be an important target to help reduce the SUID risk.

Caregivers who reported household tobacco use practiced prone sleeping, bed-sharing, and soft bedding use significantly more often compared to caregivers in households without tobacco use. Some studies have highlighted the additive SUID risk of bed-sharing and smoking, with non-smoking bed-sharers having a 1.66 increased odds of SUIDs while smoking bed-sharers having a 6.27 increased odds (Vennemann et al. 2012). However, to our knowledge, few studies have assessed smoking with prone sleeping and/or soft bedding use. Additionally, although several studies have documented why caregivers do not practice safe sleep, few have explored why tobacco users or households with tobacco use are less likely to practice safe sleep. Possibilities for these findings include that those who practice risky health behaviors are more likely to practice other risky health behaviors. Similarly, a safe sleep study in Sweden found that mothers who did not follow infant supine sleeping recommendations were less likely to follow other infant care recommendations (Lindgren et al. 1998).

Additionally, we found that our caregivers seem to practice these three safe behaviors the same or slightly more often than the general population. We hypothesize that this may be partly due to their participation in a home visiting program. According to Illinois’ 2013 Pregnancy Risk Assessment Monitoring System data, 77.5% of mothers practiced back sleeping compared to 85% of our MIECHV caregivers (2013 Illinois PRAMS Data - Infant Care 2013). In regards to bed-sharing, data from the National Infant Sleep Position Study from 2001 to 2010 found that 46% of caregivers discussed bed-sharing in the preceding 2 weeks while slightly more (49%) MIECHV caregivers bed-shared (Colson et al. 2013). For soft bedding use, the National Infant Sleep Position Study from 2008 to 2010 found that soft bedding use was around 55% while only 35% of our caregivers used soft bedding (Shapiro-Mendoza et al. 2015). Many of our caregivers were also breastfeeding and not bed-sharing, a result that follows AAP recommendations, but is contrary to the norm. We believe these results could be in part due to home visiting programs, which have been shown to be effective in improving health outcomes for at-risk families (Avellar and Supplee 2013). Illinois MIECHV home visitors regularly visit caregivers, and studies have shown that safe sleep behaviors may be improved through frequent messaging (Smith et al. 2016). It may be possible that home visitors in our population provide safe sleep education to caregivers during multiple visits, leading to higher safe sleep adherence; however, no universal safe sleep training existed at the time of this analysis, and the uniformity and extent to which home visitors are delivering safe sleep messaging is unknown.

With the understanding and analysis of baseline data, Illinois MIECHV is currently working with local non-profits to develop customized safe sleep trainings for home visitors. The first safe sleep training occurred in June 2017, and some resources used during these seminars can be found on Illinois MIECHV’s website (igrow Illinois Home Visiting Program 2017). Illinois MIECHV will continue monitoring caregivers’ safe sleep adherence levels and refine the safe sleep trainings as needed.

Limitations of this study are acknowledged. Although we analyzed a large number of MIECHV caregivers who responded to the safe sleep survey, we did not determine how many eligible caregivers did not complete this survey. Therefore, it is unclear whether our results are representative of the entire MIECHV population. Our data are also not generalizable to the overall population due to our population being from at-risk communities, and replication of our findings are needed. Additionally, all caregiver responses were self-reported to the home visitors, and home visitors manually entered the responses into an online system. Home visitors did not observationally validate these findings. However, the well-understood bias associated with self-report or survey data is likely no greater than other studies of safe sleep practices, as almost all safe sleep studies use a survey methodology to collect data. Specifically, caregivers may under-report unsafe sleep practices because of the stigma associated with not practicing safe sleep (Ward 2015).

## Conclusions

Breastfeeding caregivers in this study bed-shared less often than their non-breastfeeding counterparts. While the reasons for breastfeeding without bed-sharing are multifactorial, this study calls into question the commonly held assumption that bed-sharing is necessary for successful breastfeeding. Demonstrating the feasibility of breastfeeding and practicing safe sleep in this high SUID risk population has implications toward the possibility of reducing SUID risk in similar populations through both promoting breastfeeding and discouraging bed-sharing. This study also demonstrated the confluence of risk factors present in households with tobacco use, where caregivers adhered to safe sleep guidelines less often. Understanding that an infant living with a tobacco user is less likely to be sleeping safely suggests that a multifaceted approach to safe sleep counseling may be needed for such families. Finally, caregivers participating in these Illinois MIECHV-supported home visiting programs demonstrated greater adherence to safe sleep guidelines than expected. This highlights the importance, success, and ongoing opportunity of the health promotion role that home visitors play in the lives of the women and children receiving their critical and comprehensive services.

## Abbreviations

AAP: The American Academy of Pediatrics
MIECHV: Maternal, Infant, and Early Childhood Home Visiting Program
SIDS: Sudden infant death syndrome
SUID: Sudden unexpected infant death
